# An autosomal recessive leucoencephalopathy with ischemic stroke, dysmorphic syndrome and retinitis pigmentosa maps to chromosome 17q24.2-25.3

**DOI:** 10.1186/1471-2350-13-18

**Published:** 2012-03-21

**Authors:** Ahmed Bouhouche, Ali Benomar, Leila Errguig, Lamiae Lachhab, Naima Bouslam, Jehanne Aasfara, Sanaa Sefiani, Layachi Chabraoui, Elmostafa El Fahime, Abdeljalil El Quessar, Mohamed Jiddane, Mohamed Yahyaoui

**Affiliations:** 1 Service de Neurologie et de Neurogénétique, Hôpital des Spécialités, Rabat, Morocco; 2 Faculté de Médecine et de Pharmacie, Université Mohamed V Souissi, Rabat, Morocco; 3 Service de Neurophysiologie clinique, Hôpital des Spécialités, Rabat, Morocco; 4 Service d'anatomopathologie, Hôpital des Spécialités, Rabat, Morocco; 5 Laboratoire de Biochimie, Hôpital d'Enfants, Rabat, Morocco; 6 Plateforme génomique fonctionnelle, Unité d'Appui Technique à la Recherche Scientifique, CNRST, Rabat, Morocco; 7 Hopital Universitaire International Cheikh Zaïed, Rabat, Morocco; 8 Service de Neuroradiologie, Hôpital des Spécialités, Rabat, Morocco

## Abstract

**Background:**

Single-gene disorders related to ischemic stroke seem to be an important cause of stroke in young patients without known risk factors. To identify new genes responsible of such diseases, we studied a consanguineous Moroccan family with three affected individuals displaying hereditary leucoencephalopathy with ischemic stroke, dysmorphic syndrome and retinitis pigmentosa that appears to segregate in autosomal recessive pattern.

**Methods:**

All family members underwent neurological and radiological examinations. A genome wide search was conducted in this family using the ABI PRISM linkage mapping set version 2.5 from Applied Biosystems. Six candidate genes within the region linked to the disease were screened for mutations by direct sequencing.

**Results:**

Evidence of linkage was obtained on chromosome 17q24.2-25.3. Analysis of recombination events and LOD score calculation suggests linkage of the responsible gene in a genetic interval of 11 Mb located between D17S789 and D17S1806 with a maximal multipoint LOD score of 2.90. Sequencing of seven candidate genes in this locus, *ATP5H, FDXR, SLC25A19, MCT8, CYGB, KCNJ16 *and *GRIN2C*, identified three missense mutations in the FDXR gene which were also found in a homozygous state in three healthy controls, suggesting that these variants are not disease-causing mutations in the family.

**Conclusion:**

A novel locus for leucoencephalopathy with ischemic stroke, dysmorphic syndrome and retinitis pigmentosa has been mapped to chromosome 17q24.2-25.3 in a consanguineous Moroccan family.

## Background

Nuclear single-gene disorders related to ischemic stroke are an important cause of stroke, especially in young patients without known risk factors [1]. These diseases could be associated with different stroke phenotypes including arterial dissection, small vessel, and large vessel which are inherited as dominant, recessive or X linked trait. Up to date, 4 loci have been described for the autosomal dominant form. Cerebral Autosomal-Dominant Arteriopathy with Subcortical Infarcts and Leukoencephalopathy (CADASIL) which is a generalized disease of the small arteries, largely predominating in the brain caused by mutations in *NOTCH3 *gene [2,3]. Hereditary endotheliopathy with retinopathy, nephropathy, and stroke (HERNS) is a rare multisystemic disease presenting with leukoencephalopathy, progressive visual loss and nephropathy due to mutations in the *TREX1 *gene [4]. Marfan syndrome (MFS) which is a connective tissue disorder with multisystemic manifestations involving skeletal, cardiovascular and ocular systems caused by mutations in a very large gene *FBN1 *[5]. The Ehlers-Danlos syndrome type IV (EDS) is a connective tissue disorder defined by characteristic facial features, translucent skin, easy bruising, and severe arterial, digestive and uterine complications resulting from mutations in *COL3A1 *gene [6].

In addition to the autosomal dominant forms, three autosomal recessive forms have been also reported. Cerebral autosomal recessive arteriopathy with subcortical infarcts and leukoencephalopathy (CARASIL) is a cerebral small vessel disease accompanied by alopecia and spondylosis caused by mutations in the HTRA1 gene [7]. Pseudoxanthoma elasticum (PXE), associated with stenotic lesions of the distal carotid artery and with small vessel disease, is characterized by skin, eyes, and cardiovascular complications and related to mutations in the ABCC6 gene on chromosome 16p13.1 [8]. The homocystinuria is characterized by developmental delay/intellectual disability, ectopia lentis and/or severe myopia, skeletal abnormalities and thromboembolism caused by mutations in the CBS gene [9].

Fabry disease (FD) is a progressive X-linked inherited disorder of glycosphingolipid metabolism due to deficient or absent lysosomal alpha-galactosidase A activity. FD is associated with Cerebrovascular symptoms due to the damage of small and large blood vessels, progressive renal failure, cardiac disease, small-fiber peripheral neuropathy, and skin lesions caused by mutation in the *GLA *gene at Xq22 [10].

Finally, Moyamoya disease (MMD), characterized by bilateral stenosis and/or occlusion of the terminal portion of the internal carotid artery, could be inherited as dominant, recessive and X-linked trait [11,12]. Several genetic loci have been reported on chromosomes 3p, 6q and 17q but none of the relevant genes have yet been identified [13-16].

In the present study, we report on a consanguineous Moroccan family with three patients displaying an autosomal recessive leucoencephalopathy with ischemic stroke, dysmorphic syndrome and retinitis pigmentosa. The genome wide search mapped this new clinical entity to a new locus on chromosome 17q24.2-25.3 in a chromosomal segment of 11 Mb.

## Methods

### Patients

**Figure 1:**
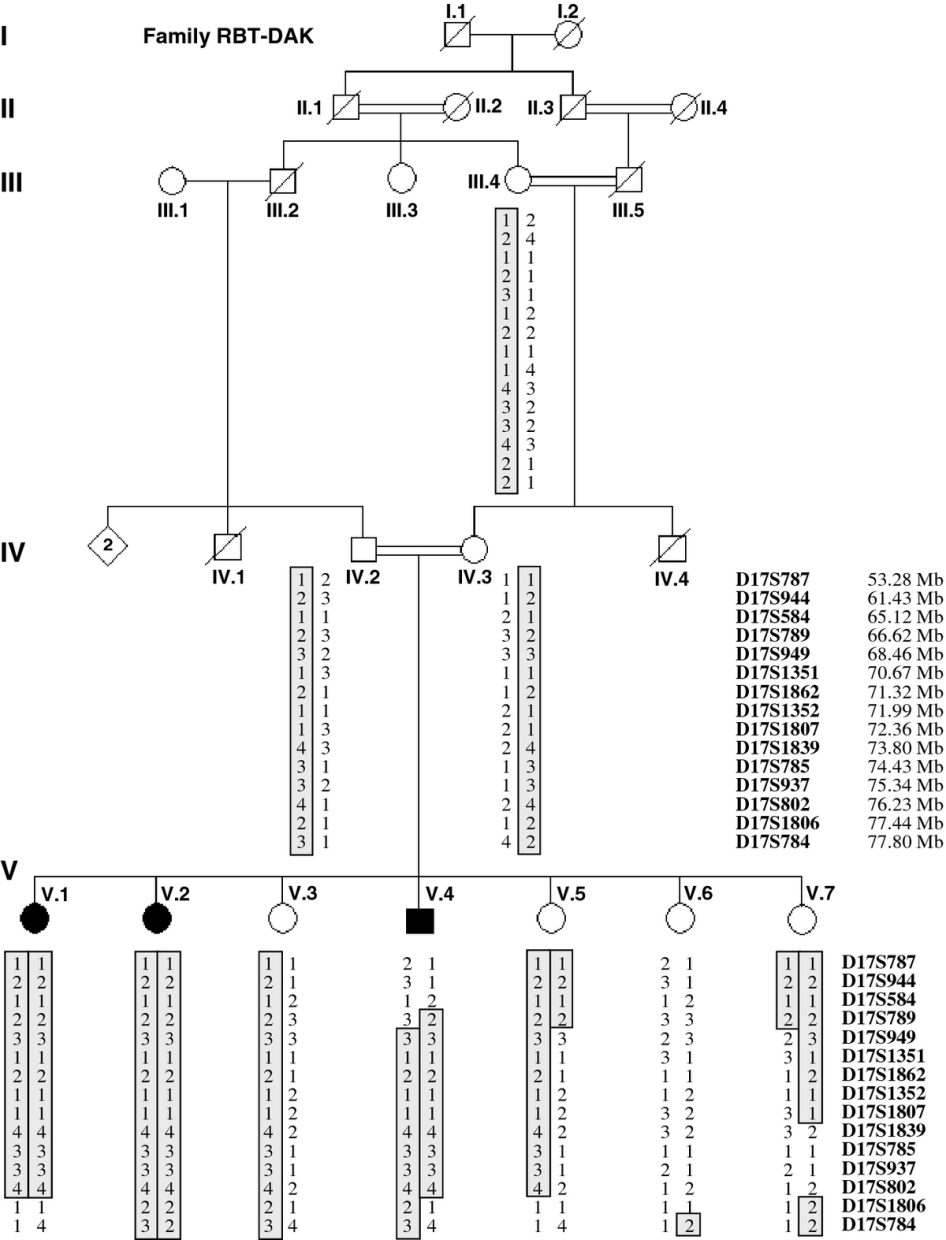
**Haplotype reconstruction for chromosome 17q in pedigree RBT-DAK**. Microsatellite markers are ordered according to the ensembl genetic map from centromere (top) to telomere (bottom). The hatched area represents the homozygosity region for each individual.

A consanguineous family of Moroccan Origin (RBT-DAK) was examined by neurologists from the Department of Neurology and Neurogenetics, Hôpital des Spécialités, Rabat, Morocco (Figure 1). Blood samples were taken from patients and family members and genomic DNA was extracted from peripheral blood leukocytes using a standard phenol/chloroform method. Brain MRIs were obtained for the three patients (V.1, V.2, and V.4) with a high field strength system (General Electric Sigma Excite2 1.5 Tesla System head coil antenna). The MRI protocol included axial T2-weighted images, sagittal T1-weighted, coronal T2-weighted, fluid attenuated inversion recovery and axial diffusion-weighted images. The MR spectroscopy, muscle biopsy and cardiac exam were done in index patient V.2 and MR angiography (MRA) and standard karyotype were done in patient V.4. All the studies were carried out after approval of the Moroccan ethical committee of biomedical research (CERB). Patient tutors signed the consent form and gave permission to publish their clinical data.

### Genotyping and linkage analysis

A genome-wide search was conducted using the ABI PRISM linkage mapping set version 2.5 from Applied Biosystems. The set consists of 400 fluorescent microsatellite markers, selected from the Généthon human linkage map covering the entire human genome with a resolution of approximately 10 cM. These markers were PCR amplified according to the manufacturer's instructions. PCR products of each panel were then pooled, combined in a tube with GeneScan 500LIZ size standard and loaded in an automated capillary DNA sequencer ABI 310. Data were collected and analysed using the ABI GENESCAN (version 2.1) and GENOTYPER (version 2.0) software (Applied Biosystems, Foster City, CA). The C.E.P.H. 1347-02 DNA was used as control.

On the basis of the segregation pattern observed in the studied family, pairwise and multipoint LOD scores were calculated using the Allegro v1.2c2 software [17], assuming a fully penetrant autosomal recessive trait with a disease allele frequency of 0.00005, 0% phenocopy and equal recombination fraction in males and females. Haplotypes were constructed according to the principles of Thomson (1987). The order and distance between markers were that of the ensembl.org genetic map.

### Sequencing of candidate genes

The 5' and 3' UTRs and all the coding exons, including exon-intron boundaries, of seven candidate genes were amplified by PCR and both strands were sequenced using Big Dye Terminator Cycle Ready Reaction Kits (Applied Biosystems) on an ABI 310 sequencer. Sequence chromatograms were analyzed using SeqScape software version 2.1 (Applied Biosystems).

## Results

### Clinical description

In the family we investigated here, the healthy parents and grand-parents of the affected siblings were first cousins and representing one of the four consanguinity loops in the pedigree. Thus, the segregation of the disease was in favor of an autosomal recessive mode of inheritance.

**Figure 2:**
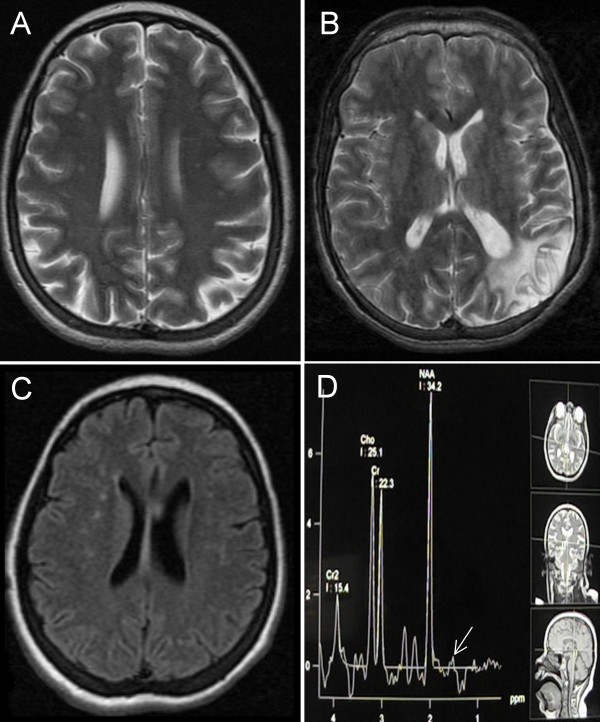
**MRI and MR spectroscopy of RBT-DAK patients**. Axial T2-weighted images of patients V.1 (A) and V.4 (B), FLAIR image of patient V.2 (C) and MR spectroscopy of patient V.2 (D) showing a small peak of lactate (see arrow).

Patient V.1, a 36-year-old female, was a full-term baby girl with no history of a neonatal suffering or infection. She had a generalised hypotonia and late acquisition of walking and talking at age of 3 years. At the age of 20, she presented progressive choreic movements, generalised myoclonus and behavioural troubles. She suffered from headache with bilateral visual loss but with no other signs. Neurological exam at age 36 years found mental retardation, pyramidal syndrome, strabismus and ocular pursuit trouble. Dysmorphic syndrome, including an oblong face, hypertelorism, arched palate, dorsal scoliosis, pes cavus, and equinovarus, was noted. Ophthalmologic exam found papillary pallor and fine vessels with retinitis pigmentosa. Brain MRI showed multiple focal white-matter hyperintensities (Figure 2A). EMG found normal motor and sensitive conductions and EEG found a normal background activity with no epileptic abnormalities. Amino acid chromatography showed an elevation of blood levels of glutamine, glycine, alanine and glutamate and elevated levels of urinary glycine and basic amino acids. Blood investigations showed normal lactate (179 mg/l) and cortisol (3.4 mg/dl). Metanephrine, normetanephrin, ACTH, ApoA and ApoB were also normal.

Patient V.2, a 33-year-old female, was a full-term baby girl with no history of a neonatal suffering or infection as well. Since the age of 5, she presented late psychomotor acquisitions and abnormal crawling movements of both arms. She didn't have any seizure or motor loss. Neurological exam at age 33 years showed mental retardation, neck dystonia, and dysmorphic syndrome with an oblong face, hypertelorism, arched palate, macroglossia and pes cavus. Cardiac exam found a heart murmur of mitral regurgitation and a hepatojugular reflux with hepatomegaly. Ophthalmologic exam found myopic chorioretinal degenerative lesions, with papillary pallor and fine vessels, and retinitis pigmentosa. Axial T2-weighted and FLAIR images showed multiple Focal white-matter hyperintensities (Figure 2C) and MR spectroscopy performed at different levels showed a small peak of lactate (Figure 2D). ENMG was difficult to perform but showed no signs of neuropathy. EEG found a normal background activity with no epileptic abnormalities. ECG showed a left ventricular hypertrophy with regular sinusal rhythm and normal echocardiography. Morphological analysis of muscle-biopsy showed no signs of atrophy or dystrophy. The histochemical analysis (NADH, SDH and COX) were normal and no red ragged fibers were seen. Blood analysis showed normal level of lactate at 181 mg/l. Cortisol (4.8 μg/dl). Electrolytes, urea, creatinin, hepatic enzymes were also normal. Amino acid chromatography showed elevated blood levels of glutamine, glycine, alanine and glutamate and elevated levels of urinary glycine and basic amino acids.

Patient V.4, a 27-year-old male, presented at the age of 7 months a fever and dystonic movements. He had late psychomotor development, as he walked at age of 7 years and did not talk; he also had an autistic behaviour. At the age of 17, he had several generalised seizures. Neurological exam at age 27 years found mental retardation, generalised dystonia, and pyramidal syndrome, along with a dysmorphic syndrome including hypertelorism, arched palate and dorsal scoliosis. Brain CT scan showed a left systemised temporoparietal hypodensity and another right temporo-occipital hypodensity evoking sequellar vascular lesions (data not shown). Axial T2-weighted and FLAIR images at different levels showed a sequellar ischemic stroke of the left posterior sylvian territory (Figure 2B). The MRA was normal and did not show signs of Moyamoya disease. Blood investigations found low level of cholesterol 1.41 g/l (normal 1.50-2.50) and normal level of cortisol (6.2 μg/dl). Metanephrin (0.08 mg/24 h), normetanephrin (0.17 mg/24 h), corticotrophin (17.9 ng/l), ApoA1 (0.84 g/l) and ApoB (0.53 g/l) were also normal. He presented several episodes of severe hypochromic microcytic anemia which required blood transfusion. Amino acid chromatography showed an elevation of blood levels of glutamine, glycine, alanine and glutamate and elevated levels of urinary glycine and basic amino acids. Chromosomal analysis showed a normal karyotype in the 11 observed metaphase cells.

### Mapping and sequencing of candidate genes

**Figure 3:**
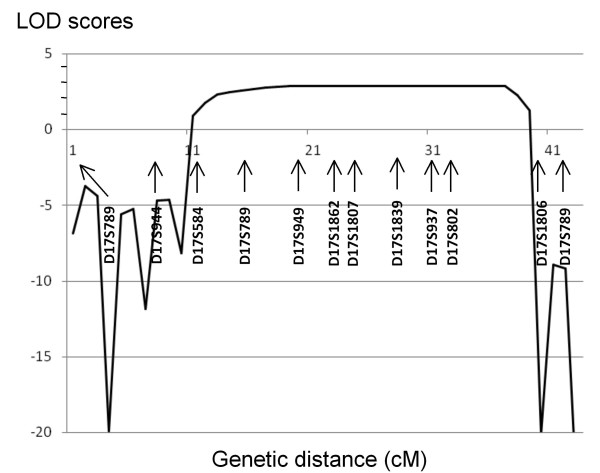
**Multipoint linkage analysis in family RBT-DAK**. The genetic distances (in cM) are represented as on the ensembl.org chromosome 17 map.

Since no similar clinical phenotype has been reported so far in the literature, a genome wide search was directly conducted in all members of family RBT-DAK. Pairwise LOD scores were negative (< -2) for all chromosomes except markers in chromosomes 9, 14 and 17, and significant scores were obtained only with D9S285, D14S258 and D17S785 markers. The two locations on chromosomes 9 and 14 were excluded by using 15 additional microsatellites which gave negative multipoint LOD scores (data not shown). Sixteen additional markers closely linked to D17S785 were used to analyze a 24 Mb of the 17q interval flanked by D17S787 and D17S784. Nine consecutive markers in the D17S789-D17S1806 interval generated positive bi-point LOD scores at θ = 0 (Table 1). The highest scores were obtained for D17S1807 (Z = 2.8910). A maximal multipoint LOD score of 2.90 was obtained for the genetic interval between D17S1862 and D17S802 (Figure 3). As the family studied is highly inbred with many consanguinity loops, the expected linked region must be large of several cM. Therefore, evidence of linkage takes into account the LOD score values and the analysis of the haplotype in the family. Haplotype analysis showed large homozygosity regions in all patients at this 17q24-25 location. Recombination events between D17S789 and D17S949 in individual V.7 and between D17S802 and D17S1806 in individuals V.1 and V.4 defined a new possible locus of approximately 11-Mb between markers D17S789 and D17S1806 (Figure 1). Note that the healthy individual V.5 is homozygous for D17S949 and D17S1351 but her mother IV.3 is not informative for these two markers.

**Table 1:** Two point LOD score for microsatellite markers of family RBT-DAK calculated at recombination fraction θ = 0

Markers	Position (Mb)	θ = 0.00
D17S787	53,282,084	- ∞

D17S944	61,436,306	- ∞

D17S584	65,119,869	- ∞

D17S789	66,628,565	- ∞

D17S949	68,465,446	1.2268

D17S1351	70,672,152	1.1828

D17S1862	71,326,581	2.4988

D17S1852	71,998,011	1.3985

D17S1807	72,360,592	2.8910

D17S1839	73,801,863	2.8469

D17S785	74,431,373	2.6879

D17S937	75,347,271	2.4608

D17S802	76,234,610	2.6879

D17S1806	77,445,675	- ∞

D17S784	77,802,191	- ∞

This new genetic locus contains ≈ 140 known genes according to the genome database (Ensembl.org). The large clinical spectrum of the disease in the family RBT-ENC and the presence of small peak of lactate found in MR spectroscopy, make the three genes that encode proteins with known mitochondrial functions strong candidate for the disease. These include *ATP5H, FDXR *and *SLC25A19 *encoding ATP synthase subunit d, ferredoxin-NADP (+) reductase and solute carrier family 25 (mitochondrial thiamine pyrophosphate carrier), member 19 respectively. Since mutations in *MCT8 *gene lead to a syndrome of severe psychomotor retardation [18], we also sequenced *MCT6 *encoding the monocarboxylic acid transporter 7. We next analyzed the *CYGB *gene, encoding a cytoglobin which may be involved in oxygen storage and delivery and may acts as an endogenous neuroprotective factor in focal cerebral ischemia [19]. Since glutamate and N-methyl-D-aspartate (NMDA) receptors have been implicated in several disorders of the central nervous system including stroke [20] and GRIN2A and GRIN2B genes have been recently implicated in encephalopathy with mental retardation and epilepsia [21], we sequenced the *GRIN2C *gene encoding the glutamate receptor, ionotropic, N-methyl D-aspartate 2 C. We also sequenced the *KCNJ16 *gene since KCNJ2 and KCNJ10 have been associated with mental retardation, dysmorphic syndrome and a CNS phenotype [22,23]. No causative mutations were identified in the coding sequence of all these genes. However, in the FDXR gene, we found three missense mutations reported as SNP in ensembl.org data base (rs1688149 (c.275A > G/p.Asp92Gly); rs690514 which was found in 4 transcripts (c.368 G > A/Arg123Gln; c.461 G > A/p.Arg154Gln; c.497 G > A/p.Arg166Gln; c.236 G > A/p.Arg79Gln); and rs552432 (c.887 T > C/p.Leu296Pro)) at homozygous state in all patients and segregated with the disease in the family. Sequencing of 60 DNA individual controls showed that 20/60 presented this haplotype with the three mutations at heterozygous state and 3/60 at homozygous state. As the disease is considered very rare, these variants should be polymorphism rather than causal mutations.

## Discussion

Consanguineous families with rare disease are extremely useful in defining new loci for autosomal recessive diseases. In the family investigated here, the segregation of the disease was in favor of an autosomal recessive mode of inheritance. In all patients, the disease starts in early infancy by psychomotor retardation and progressive dystonic and choreic movements. At age of exam, all patients presented severe mental impairment, dysmorphic syndrome and retinitis pigmentosa. Only patient V.4 had epilepsia with several episodes of generalized seizures. Radiological investigations showed multiple ischemic lesions interesting small arteries in two patients (V.1 and V.2) and large artery in patient V.4, whereas MRA was normal in this patient. The clinical presentation was therefore associated in the studied family with leucoencephalopathy with ischemic stroke, dysmorphic syndrome and retinitis pigmentosa. Suggestive linkage of the responsible gene was found on chromosome 17q24.2-25.3 in an 11 Mb genetic interval between D17S789 and D17S1806 with a maximal multipoint LOD score of 2.90. Two loci of Autosomal dominant Moyamoya disease have been mapped to chromosome 17q. The locus of Mineharu *et al. *(2008) was distal to D17S1806 marker and thus did not overlap with our new locus. However, it overlaps with the locus of Yamauchi T *et al. *(2000) but the clinical presentation in the family studied here was different of the one reported for Moyamoya disease.

Seven candidate genes in this locus, *ATP5H, FDXR, SLC25A19, MCT8, CYGB, KCNJ16 *and *GRIN2C *were sequenced but no causative mutation was found. The clinical presentation is very broad in the family studied here and suggests that the disease could be due to a nuclear mitochondrial gene, but the lactate level was normal in blood and MR spectroscopy showed only small peak of lactate. However, the elevated levels of amino acids observed in the blood and urine rather suggest that the defective gene may be involved in pathways of intermediary metabolism. In these pathways, amino acids, when deaminated, yield α-keto acids that, directly or via additional reactions, feed into the major metabolic pathways (e.g., Krebs cycle). More families with the same disease showing linkage to this locus are needed to reduce the candidate genetic interval and thus the number of genes to be analyzed.

## Conclusions

We report here the identification of a novel locus for autosomal recessive leucoencephalopathy with ischemic stroke, dysmorphic syndrome and retinitis pigmentosa on chromosome 17q24.2-25.3, in a genetic interval of 11 Mb. Gene identification is ongoing and should increase our understanding of the causes and the physiopathology of this ischemic stroke condition.

## Competing interests

The authors declare that they have no competing interests.

## Authors' contributions

AB performed the genotyping analysis, carried out the molecular genetic studies, participated in the design of the study and its coordination and drafted the manuscript. EE helped in molecular genetic studies. NB performed DNA extraction and banking. LE, LL and JA participated in the Medical and paramedical investigations. AEQ and MJ carried out MRI, MRA and MR spectroscopy. SS performed muscle biopsies. LC carried out biochemical investigations. MY and AB carried out medical examinations, participated in the design and coordination of the study and helped to draft the manuscript. All authors read and approved the final manuscript.

## Pre-publication history

The pre-publication history for this paper can be accessed here:

http://www.biomedcentral.com/1471-2350/13/18/prepub
